# Diabetes-Related Distress Assessment among Type 2 Diabetes Patients

**DOI:** 10.1155/2018/7328128

**Published:** 2018-03-26

**Authors:** Majed O. Aljuaid, Abdulmajeed M. Almutairi, Mohammed A. Assiri, Dhifallah M. Almalki, Khaled Alswat

**Affiliations:** ^1^Taif University School of Medicine, Taif, Saudi Arabia; ^2^Diabetes and Endocrinology Center, Prince Mansoure Hospital, Taif, Saudi Arabia

## Abstract

**Background and Objectives:**

Diabetes is one of the most common chronic diseases; it is a debilitating and hard to live with. Diabetes-related distress (DRD) refers to the emotional and behavioral changes caused by diabetes. Our study aims to assess the prevalence of DRD among type 2 diabetes (T2D) patients using Diabetes Distress Scale-17 items (DDS-17) and its relation to complications and treatment modalities.

**Methods:**

A cross-sectional study of adult T2D patients with follow-up visits at the Diabetes and Endocrinology Center in Taif, Saudi Arabia, between January and July 2017. We excluded patients with other forms of diabetes, untreated hypothyroidism, and psychiatric illness. The total score of DDS-17 was calculated by summing the 17 items' results and then dividing the total by 17. If the total score was >2, then it was considered as clinically significant results (moderate distress), but if it is ≥3, then it is classified as a high distress.

**Results:**

A total of 509 T2D patients with a mean age of 58 ± 14 years were included. The majority of participants were male, married, not college educated, and reported a sedentary lifestyle. We found 25% of the screened T2D patients have moderate to high DRD. Regarding the DRD components, emotional distress was the most prevalent followed by physician-related distress. HabA1c was significantly higher in those with high combined distress and high emotional distress compared to those with mild/moderate distress (*p* = 0.015 and 0.030, resp.).

**Conclusion:**

Our study shows that DRD is a medically relevant issue that clinicians need to address. Despite observing a low prevalence of DRD compared to other studies, we found significant correlations between DRD scores and HabA1c, triglyceride levels, BMI, T2D duration, and interval between visits.

## 1. Background

Diabetes is one of the most common chronic diseases, estimated to affect more than 400 million people worldwide. Its prevalence is predicted to grow to 642 million by 2040, and it is anticipated to be the 7th leading cause of death by 2030 [1, 2]. Diabetes will affect more than 70 million in the Middle East and North African region by 2040 [1]. Saudi Arabia and Kuwait are among the countries with the highest prevalence of diabetes, estimated at 17.6% and 14.3%, respectively [1].

Diabetes is a challenging disease that is considered to be hard to live with as it encompasses a lot of restrictive instructions. The emotional distress facing people with diabetes due to such lifestyle restriction is an area of growing clinical interest [3]. The instructions given by the educator or the physician can seem to be complicated for a person from a nonmedical background, which further compounds the emotional distress of the diagnosis and necessary lifestyle changes [3, 4].

Diabetes-related distress (DRD) and diabetes burnout are terms that have been used in the literature to refer to the emotional and behavioral changes caused by diabetes and its demanded lifestyle alterations [5, 6]. Many studies have been conducted in this field using different scales on different populations. These studies have shown that there are many factors related to the presence or absence of DRD, and its severity depends on the characteristics of the population and other psychosocial factors [7]. The dangerous complications (e.g., hypoglycemia) that can result from DRD have made this issue a growing area of interest for researchers.

A relatively new scale called Diabetes Distress Scale-17 items (DDS-17) was recently developed as a brief and reliable scale that avoids the limitations and deficits of previous metrics [3]. The brief form of DDS is designed for use in clinical settings not just for research purposes [8].

This study was motivated by the scarcity of studies on DRD in Saudi Arabia. To the best of our knowledge, there have been no studies using DDS-17 in this environmental context. Our study aims to assess distress among type 2 diabetes (T2D) patients using DDS-17 and to correlate this DRD to T2D complications, treatment modalities, and glycemic control.

## 2. Methods

Here, we present a cross-sectional study on T2D patients of the Prince Mansour Hospital, Diabetes, and Endocrinology Center in Taif City, Saudi Arabia, between January and July 2017. All T2D patients were 18 years of age or older, and all recent laboratory results were included. Patients with type 1 diabetes (T1D), untreated hypothyroidism, gestational diabetes, cancer, psychiatric illness, and patients unwilling to participate were excluded.

Researchers reviewed files for the potential patients in clinics and made sure that they fulfilled the inclusion criteria. Data collection forms (DCFs) were compiled during patient interviews using a drug picture chart to help them to identify their treatment correctly. The DCF is composed of three sections: demographic data, past medical history, and drug history. Past history includes history of severe hypoglycemia, which is defined as a decrease of blood sugar less than 55 mg/dl with loss of conscious or/and required help from someone else due to their condition in the last 12 months. Laboratory results were collected from patients' electronic medical records. Patient records were anonymized by replacing patient name and medical record number (MRN) with a serial study code prior to export into a statistical analysis program. Our research proposal was reviewed and approved by the Institutional Review Board (IRB) and referenced by H-02-001-16-11-256.

We used DDS-17 to assess DRD in study participants. Each of the 17 items of DDS-17 has a six-point scale for response: a mild to moderate problem is 1 or 2, a moderate to serious problem is 3 or 4, and a serious problem is 5 or 6. The total score of DDS-17 was calculated by summing the 17 items' results and dividing by 17. If the total score was >2, then it was considered as clinically significant results (moderate distress), but if it is ≥3, then it is classified as a high distress [3]. DDS-17 assesses four components of DRD, which are emotional, physician-related, regimen-related, and interpersonal distress. Each component scored separately by dividing the sum of its item scores by the number of the items. Patients were considered to have DRD in each component separately if they scored >2 in that component.

DDS-17 items were translated to the Arabic language by one bilingual person and validated by another 2 bilingual experts, and it was tested on a pilot sample (100) showing good internal consistency for the whole 17 items as shown in Table 1 (Cronbach's alpha = 0.877).

We could not calculate the sample size of this study due to the absence of an official number of people with T2D in this country. So, we decide to calculate the required sample size based on the approximation process knowing that the IDF estimating the DM in Saudi Arabia by 3.85 million (latest estimation) and the number of population in Saudi Arabia was 32.6 million and 556,100 in Taif City. 68,496 was the estimated number of people with diabetes mellitus in Taif City. 596 was the targeted sample size based on the approximation process (95% CI, 5% margin of error). This number was not corrected for the people with T1D due to the scarcity of information about the burden of this disease. The number of people who were attending the center for their appointment in the diabetes clinics (only 2) was 30 on average (scheduled). This is why we have chosen the convenience sampling method for a specific period (1 month) and included all the people who have an appointment in the center and fulfill the inclusion criteria and intact from the exclusion criteria. Extending the period was done by month in case it was needed till we have reached what was calculated based on the approximation. The extension of the period was due to many reasons:

1. Discrepancy between the average number and the real number due to many reasons (e.g., vacation)
2. Significant number of those who have follow-up visits were T1D
3. High number of those who have other exclusion criteria

The number of people who were recruited was 604, and 509 was the final number after excluding those with missing values in the DDS-17.

For analysis, we used Statistical Package for the Social Sciences (SPSS) program v20. All continuous variables were expressed as mean ± SD. Categorical variables were compared with the *χ*^2^ test and Student's *t*-test for comparing means of continuous variables. One-way ANOVA was used to compare ≥3 means against an independent variable. A *p* value must be less than 0.05 to be considered statistically significant.

## 3. Results

A total of 509 T2D patients with a mean age of 58 ± 14 years were included. The majority of participants were male, married, not college educated, and reported a sedentary lifestyle (Table 2).

Analysis of DDS-17 results indicated that 25% of our sample have moderate to high DRD based on the total score of the questionnaire. In addition, 54% of our samples have moderate to high emotional distress, 24.9% have moderate to high physician-related distress, 12.7% have moderate to high regimen-related distress, and 7.7% have moderate to high interpersonal distress.

Examining the differences between the highly distressed and moderately/mildly distressed groups showed significant relationships between the score and age, body mass index (BMI), blood pressure (BP), duration of diabetes, interval between visits of a doctor, and laboratory results (Tables 3–7). Regarding laboratory results, HabA1c was significantly higher in those with high combined distress and high emotional distress compared to those with mild/moderate distress (*p* = 0.015 and 0.030, resp.). Additionally, LDL was significantly higher in the group with high combined distress compared to that with mild/moderate combined distress (*p* = 0.027). Longer T2D duration was associated with emotional, physician-related, and regimen-related distress (*p* = 0.034, 0.14, and 0.026, resp.). Similarly, the younger patients are more liable to have combined and regimen-related distress (*p* = 0.027 and <0.0001, resp.).

The means of the total and individual component scores of DDS-17 were analyzed separately (Table 8). Using DDS-17 total scores, we found subjects were more likely to have DRD who were females (*p* < 0.0001), were low income (*p* = 0.047), were unemployed (*p* < 0.0001), have any diabetes complications (*p* < 0.0001), have retinopathy (*p* < 0.0001), have neuropathy (*p* = 0.015), have dyslipidemia (*p* = 0.025), have a family history of T2D (*p* = 0.052), had severe hypoglycemia (*p* = 0.006), and have a history of T2D-related hospital admission (*p* = 0.001).

Those with emotional distress were significantly more likely to be female (*p* < 0.0001), have high income (*p* = 0.019), unemployed (*p* < 0.0001), insulin and oral hypoglycemic drug users (*p* = 0.006), have any diabetic complications (*p* < 0.0001), have retinopathy (*p* < 0.0001), have neuropathy (*p* < 0.0001), have dyslipidemia (*p* = 0.002), have hypertension (*p* = 0.028), have a T2D family history (*p* < 0.0001), have a history of severe hypoglycemia (*p* = 0.04), and have previous T2D-related hospital admissions (*p* < 0.0001).

Physician-related distress score was significantly associated with low income (*p* = 0.011), unemployment (*p* = 0.038), and a history of severe hypoglycemia (*p* = 0.018). In addition, treatment regimen distress was associated with having been divorced (*p* < 0.0001), employed (*p* = 0.001), smoker (*p* = 0.007), insulin and oral hypoglycemic drugs users (*p* = 0.03), peripheral vascular disease (*p* = 0.018), and nephropathy (*p* = 0.015). Nevertheless, those who were female (*p* = 0.002), physicians (*p* = 0.015), unemployed (*p* = 0.004), without a nephropathy history (*p* = 0.021), experienced severe hypoglycemia (*p* = 0.016), and did not have previous T2D-related hospital admissions (*p* = 0.043) were more likely to have high DRD in the interpersonal domain. Also, *p* values from the one-way ANOVA tests to assess the differences between groups vertically and horizontally are listed in Table 8. Most of the *p* values horizontally were significant due to the big differences between emotional distress and the other scores.

Partial correlation adjusting for gender, marital status, level of education, socioeconomic status, employment, treatment modalities, and exercise showed significant correlations between DRD total score and/or DRD component scores with HabA1c, triglyceride levels, BMI, T2D duration, and interval between visits (Table 9).

## 4. Discussion

Our study showed that around 25% of the participants screened positive for moderate to high DRD on a DDS-17 scale. A study that was done in the USA using the DDS-17 scale showed that 51.3% of the screened participants have moderate to high DRD [9] Another study in Malaysia revealed that about 49.2% of their T2D population has moderate distress on a DDS-17 scale [10]. Similarly, distress proportions were 48.5%, 43%, and 39% in three different studies using DDS-17 from Bangladesh, China, and Canada, respectively [11–13]. On the other hand, two studies from Germany used Problem Areas in Diabetes Questionnaire (PAID) to show that 8.9% and 10.7% of their sample were distressed [14, 15].

This discrepancy between the previously reported DRD proportions and the DRD prevalence in this study might result from different assessment tools since some of them used PAID. However, for those that used DDS-17, there are many variables that might explain this variation, including big differences between sample sizes. Easy accessibility to the health care centers and free replenishment of drugs could explain the observed lower prevalence in our study. In particular, this issue was depicted in this study as the lowest mean of the DDS-17 scores being for physician-related and regimen-related distress after interpersonal distress, while the highest score was emotional.

Demographic data of the participants was useful towards determining the group most affected by DRD. Our study showed many results in line with other studies' results regarding demographic variables (marital status, level of education, income, and employment). Our study showed that marital status was a significant differential factor in one domain (regimen-related) while in a study done in Iran, it correlated with significant differences in all domains of DDS-17 except interpersonal [16]. In our study, the level of education of the participant and the choice of a physician was assessed. These additions proved advantageous as our study revealed that the physician group was a source of high distress on all domains compared to the other groups. Also, lower education level was associated with more distress as reported in the previous study [11]; this finding was similar to ours if we exclude the participated physician. In our study, the low-income group was more affected in two domains only of DRD (physician-related and interpersonal). This finding was not in line with a previous study that showed low-income patients were more affected by total distress [11]. The unemployed participants were more likely to have DRD in this study, as found in previous studies [9, 11]. Our study showed an association between DRD and its components and smoking status, duration of T2D, complications, BMI, management method, and glycemic control. This finding endorsed similar findings from previously published studies [4, 9, 10, 16, 17].

Similar to previous studies, our study showed that the duration of diabetes was significantly associated with DRD and three of its domains (emotional, physician-related, and regimen-related) [3, 11]. This finding is corroborated by two other studies that also found duration to be significantly associated with DRD [3, 11]. Although our study showed a positive correlation between the intervals of the follow-up visits and DRD, this issue was not examined by previous studies to the best of our knowledge.

Our study showed a positive correlation between both the DRD total score and emotional distress with the HabA1c. As expected, this finding was linear to the previous studies' results. [10, 11, 14, 16, 17]. Previous studies have shown a significant correlation between DRD and depression and anxiety [9, 10, 17]. Although we did not screen for psychological factors, they could be related to our observations. It has been shown that improving depressive symptoms has a beneficial effect on HabA1c [18]. However, this effect is mild compared to the effect of improving distress on HabA1clevels, as shown in the interventional study done by Kuniss et al. [19]. This study showed that involving subjects in an educational program at a diabetes clinic can improve glycemic control and correlated with a decrease in DRD. An additional interventional study examined the possibility of improving depressive symptoms in patients with improved DRD, and the authors concluded that DRD is an independent factor that may have an effect on depression [20].

DRD can be measured by both self-reporting methods and biological methods. Biological methods include measuring salivary alpha-amylase, which is considered a good indicator of stress, as it is an indicative of sympathetic nervous system activity. Both methods were used in a study that examined the relationship between DRD and cardiovascular disease (CVD) and concluded that there was a significant relationship between DRD and CVD [21]. These findings are in line with our findings that DRD correlated with worse cardiovascular markers.

This project has three limitations. Firstly, the absence of a previous study in the field renders ours hard to compare to others. Secondly, we were limited by the absence of previous community-based studies that document the number of T2D patients towards determining the sample size for any future projects. Thirdly, this is a single-center study and we believe a multicenter one is required to investigate this problem nationwide, similar to the Diabetes Attitude, Wishes and Needs (DAWN) projects [22, 23].

## 5. Conclusions and Recommendations

Our study shows that DRD is a medically relevant issue that clinicians need to address to successfully manage T2D. The associations found in this study mirrored those found in previous studies and further endorsed the need for clinical attention to DRD, especially in societies with a high prevalence of T2D. This study demonstrates a low prevalence of DRD compared to the majority of other studies. We observed significant correlations between DRD total score and/or DRD components scores with HabA1c, triglyceride levels, BMI, T2D duration, and interval between visits. DRD has an effect on glycemic control as shown in this study and previous studies [10, 11, 14, 16, 17] So, it is a considerably important issue that has to be taken in caring the people with diabetes. The suggested management strategies are as follows:

1. Screen for the DRD by the 2-item DDS.
2. Screen the 4 components if it is needed (distressed patients on the DDS-2) by the DDS-17.
3. Offer help for them and engage their family into the management plan and exclude other causes for distress (if known from the past medical history).
4. Management plan: this includes boosting the self-care practices and coping skills [24].
5. Monitor the glycemic control by the HabA1c (if needed as a baseline).
6. Monitor the adherence to the newly applied methods that were used to alleviate the DRD.
7. Screen them every 3 months accompanied by HabA1c (if it was affected in the last visit).

Also, we recommend future interventional studies to be made, in order to determine the best approaches for a physician to prevent and treat distressed patients and to apply these approaches in our Eastern society.

## Figures and Tables

**Table 1:** Internal consistency coefficient (Cronbach's alpha) values for Diabetes Distress Scale-17 items (DDS-17).

DDS-17 items	Cronbach's alpha if item deleted
1. Feeling the diabetes is taking up too much of my mental and physical energy every day	0.868
2. Feeling that my doctor doesn't know enough about diabetes and diabetes care	0.877
3. Feeling angry, scared, and/or depressed when I think about living with diabetes	0.861
4. Feeling that my doctor doesn't give me clear enough directions on how to manage my diabetes	0.874
5. Feeling that I am not testing my blood sugars frequently enough	0.873
6. Feeling that I am often failing with my diabetes routine	0.868
7. Feeling that friends or family are not supportive enough of self-care efforts (e.g., planning activities that conflict with my schedule, encouraging me to eat the “wrong” foods)	0.870
8. Feeling that diabetes controls my life	0.874
9. Feeling that my doctor doesn't take my concerns seriously enough	0.875
10. Not feeling confident in my day-to-day ability to manage diabetes	0.868
11. Feeling that I will end up with serious long-term complications no matter what I do	0.863
12. Feeling that I am not sticking closely enough to a good meal plan	0.870
13. Feeling that friends or family don't appreciate how difficult living with diabetes can be	0.871
14. Feeling overwhelmed by the demands of living with diabetes	0.871
15. Feeling that I don't have a doctor, who I can see regularly enough about my diabetes	0.875
16. Not feeling motivated to keep up my diabetes self-management	0.872
17. Feeling that friends or family don't give me the emotional support that I would like	0.871

**Table 2:** Baseline characteristics for the current sample.

Baseline characteristics	Mean/percentage
Age	58 ± 14
DBP	75.13 ± 11.21
SBP	131.87 ± 20.72
BMI	30.89 ± 6.96
Interval between visits	4.3 ± 1.8
Duration of DM in years	14.19 ± 8.83
Gender	
Male	329 (64.6%)
Female	180 (35.4%)
Marital status	
Married	478 (93.9%)
Single	10 (2.0%)
Divorced	5 (1.0%)
Widow	16 (3.1%)
Level of education	
Illiterate	186 (36.6%)
High secondary school or less	273 (53.7%)
Postgraduate education (master or above)	45 (9.1%)
Medical graduate: doctor	3 (0.6%)
Socioeconomic status	
Low (less than 5000 SR)	153 (30.4%)
Medium (5000–15,000 SR)	319 (63.4%)
High (more than 15,000 SR)	31 (6.2%)
Employment	
Unemployed	165 (32.5%)
Employed	83 (16.3%)
Retired/housewife	260 (51.2%)
Smoking	
Yes	39 (8.3%)
No	358 (76.0%)
Former	74 (15.7%)
Physical activity	
Sedentary lifestyle	253 (50.1%)
<150 min./week	166 (32.9%)
150–300 min./week	75 (14.9%)
>300 min./week	11 (2.2%)
Management	
Lifestyle modification	5 (1.0%)
Oral hypoglycemic drugs	173 (34.1%)
Insulin use	95 (18.7%)
Insulin and oral hypoglycemic drugs	234 (46.2%)
Complications	
Yes	265 (55.4%)
No	213 (44.6%)
Ischemic heart disease	
Yes	29 (7.0%)
No	383 (93.0%)
Cerebrovascular accident	
Yes	13 (3.3%)
No	385 (96.7%)
Peripheral vascular disease	
Yes	8 (2.0%)
No	385 (98.0%)
Retinopathy	
Yes	238 (45.9%)
No	281 (54.1%)
Nephropathy	
Yes	59 (13.3%)
No	384 (86.7%)
Neuropathy	
Yes	126 (25.6%)
No	367 (74.4%)
Dyslipidemia/hypercholesterolemia	
Yes	276 (58.5%)
No	196 (41.5%)
Hypertension	
Yes	243 (54.7%)
No	201 (45.3%)
Severe hypoglycemia	
Yes	196 (38.5%)
No	313 (61.5%)
Hospital admission due to diabetes-related conditions	
Yes	79 (15.6%)
No	427 (84.4%)
HabA1c	8.61 ± 1.95
Cholesterol	4.45 ± 1.08
Triglyceride	1.60 ± 1.18
HDL	1.04 ± 0.29
LDL	2.67 ± 0.92
Microalbuminuria	21.28 ± 83.62
Total distress score	1.80 ± 0.62
Emotional distress score	2.47 ± 1.19
Physician-related distress score	1.38 ± 0.77
Regimen-related distress score	1.78 ± 0.81
Interpersonal distress score	1.26 ± 0.73

**Table 3:** The mean ± SD of age and selected clinical and laboratory variables across the mild-moderate and highly distressed groups (total domain).

Clinical/laboratory variables	Result of the total score (total domain)		
	Yes	No	*p* value
	Mean ± SD	Mean ± SD	
Age	53 ± 14	59 ± 14	0.027^∗^
Duration of DM in years	14.74 ± 8.06	14.10 ± 8.91	0.696
Weight in kg	87.07 ± 21.11	83.22 ± 17.58	0.270
Height in meter	1.597 ± 0.094	1.642 ± 0.097	0.018^∗^
BMI	34.08 ± 7.85	30.65 ± 6.93	0.013^∗^
Interval between visits	5.3 ± 1.3	4.3 ± 1.8	<0.001^∗^
DBP	74.13 ± 10.64	75.11 ± 11.29	0.647
SBP	129.13 ± 18.11	132.02 ± 20.84	0.459
HabA1c	9.74 ± 1.87	8.56 ± 1.94	0.015^∗^
Triglyceride	1.64 ± 1.50	1.59 ± 1.18	0.857
Cholesterol	4.85 ± 0.91	4.42 ± 1.07	0.126
HDL	1.07 ± 0.30	1.04 ± 0.29	0.759
LDL	3.20 ± 0.78	2.65 ± 0.91	0.027^∗^
Microalbuminuria	4.25 ± 6.63	21.77 ± 86.46	0.466

^∗^Statistically significant difference (*p* < 0.05).

**Table 4:** The mean ± SD of age and selected clinical and laboratory variables across the mild-moderate and highly distressed groups (emotional domain).

Clinical/laboratory variables	Result of the total score (emotional domain)		
	Yes	No	*p* value
	Mean ± SD	Mean ± SD	
Age	59 ± 14	58 ± 14	0.641
Duration of DM in years	15.32 ± 9.21	13.53 ± 8.60	0.034^∗^
Weight in kg	83.91 ± 17.49	83.04 ± 17.82	0.617
Height in meter	1.613 ± 0.093	1.652 ± 0.97	<0.001^∗^
BMI	32.25 ± 6.49	30.12 ± 7.13	0.002^∗^
Interval between visits	4.9 ± 1.8	4.1 ± 1.8	<0.001^∗^
DBP	75.21 ± 10.89	75.04 ± 11.42	0.873
SBP	134.05 ± 20.92	130.90 ± 20.70	0.110
HabA1c	8.94 ± 2.10	8.47 ± 1.86	0.030^∗^
Triglyceride	1.50 ± 0.98	1.64 ± 1.28	0.245
Cholesterol	4.42 ± 0.99	4.45 ± 1.11	0.817
HDL	1.05 ± 0.27	1.04 ± 0.30	0.743
LDL	2.67 ± 0.86	2.67 ± 0.94	0.996
Microalbuminuria	32.72 ± 120.64	15.65 ± 56.75	0.172

^∗^Statistically significant difference (*p* < 0.05).

**Table 5:** The mean ± SD of age and selected clinical and laboratory variables across the mild-moderate and highly distressed groups (physician-related domain).

Clinical/laboratory variables	Result of the total score (physician-related domain)		
	Yes	No	*p* value
	Mean ± SD	Mean ± SD	
Age	61 ± 15	58 ± 13	0.225
Duration of DM in years	17.69 ± 9.15	13.89 ± 8.77	0.014^∗^
Weight in kg	83.90 ± 20.00	83.49 ± 17.59	0.905
Height in meter	1.599 ± 0.095	1.642 ± 0.096	0.021^∗^
BMI	32.27 ± 6.82	30.82 ± 6.98	0.298
Interval between visits	5.0 ± 1.5	4.3 ± 1.8	0.023^∗^
DBP	72.26 ± 9.57	75.38 ± 11.32	0.113
SBP	137.57 ± 20.56	131.40 ± 20.77	0.040^∗^
HabA1c	9.18 ± 2.04	8.57 ± 1.94	0.153
Triglyceride	1.54 ± 1.24	1.60 ± 1.19	0.811
Cholesterol	4.53 ± 0.68	4.44 ± 1.10	0.563
HDL	0.98 ± 0.25	1.04 ± 0.29	0.313
LDL	2.78 ± 0.69	2.66 ± 0.93	0.576
Microalbuminuria	15.33 ± 27.70	21.16 ± 86.20	0.769

^∗^Statistically significant difference (*p* < 0.05).

**Table 6:** The mean ± SD of age and selected clinical and laboratory variables across the mild-moderate and highly distressed groups (regimen-related domain).

Clinical/laboratory variables	Result of the total score (regimen-related domain)		
	Yes	No	*p* value
	Mean ± SD	Mean ± SD	
Age	50 ± 15	59 ± 13	<0.001^∗^
Duration of DM in years	11.47 ± 7.81	14.48 ± 8.91	0.026^∗^
Weight in kg	86.52 ± 21.94	83.27 ± 17.35	0.271
Height in meter	1.636 ± 0.096	1.640 ± 0.097	0.821
BMI	31.75 ± 10.66	30.81 ± 6.52	0.586
Interval between visits	5.4 ± 1.5	4.2 ± 1.8	<0.001^∗^
DBP	76.85 ± 10.66	74.98 ± 11.29	0.279
SBP	131.28 ± 19.41	131.86 ± 20.70	0.854
HabA1c	9.19 ± 1.93	8.55 ± 1.95	0.071
Triglyceride	1.78 ± 1.34	1.57 ± 1.17	0.345
Cholesterol	4.76 ± 0.95	4.40 ± 1.09	0.071
HDL	1.12 ± 0.31	1.03 ± 0.29	0.105
LDL	2.93 ± 0.80	2.64 ± 0.93	0.098
Microalbuminuria	33.96 ± 123.04	20.00 ± 79.28	0.402

^∗^Statistically significant difference (*p* < 0.05).

**Table 7:** The mean ± SD of age and selected clinical and laboratory variables across the mild-moderate and highly distressed groups (interpersonal domain).

Clinical/laboratory variables	Result of the total score (interpersonal domain)		
	Yes	No	*p* value
	Mean ± SD	Mean ± SD	
Age	56 ± 13	58 ± 14	0.387
Duration of diabetes in years	12.39 ± 7.95	14.25 ± 8.86	0.324
Weight in kg	89.62 ± 22.32	83.11 ± 17.49	0.109
Height in meter	1.610 ± 0.075	1.640 ± 0.097	0.169
BMI	32.96 ± 11.40	30.79 ± 6.71	0.408
Interval between visits	5.3 ± 1.9	4.3 ± 1.8	0.008^∗^
DBP	73.57 ± 13.19	75.15 ± 11.07	0.506
SBP	129.61 ± 24.72	131.94 ± 20.55	0.599
HabA1c	9.42 ± 2.02	8.59 ± 1.95	0.149
Triglyceride	1.37 ± 1.68	1.61 ± 1.17	0.485
Cholesterol	4.61 ± 0.063	4.44 ± 1.09	0.637
HDL	1.05 ± 0.31	1.04 ± 0.29	0.880
LDL	3.20 ± 0.49	2.66 ± 0.93	0.085
Microalbuminuria	5.07 ± 7.73	21.83 ± 84.92	0.555

^∗^Statistically significant difference (*p* < 0.05).

**Table 8:** DDS scores and subscore mean ± SD across the demographical and clinical variables when testing these means vertically and horizontally.

Demographical/clinical variables	Total score	Emotional score	Physician-related score	Regimen-related score	Interpersonal score	*p* value
Gender						
Male	1.71 ± 0.52	2.30 ± 1.10	1.34 ± 0.69	1.75 ± 0.76	1.17 ± 0.50	<0.0001
Female	1.98 ± 0.74	2.78 ± 1.28	1.49 ± 0.92	1.87 ± 0.90	1.42 ± 1.01	<0.0001
*p* value	<0.0001	<0.0001	0.062	0.116	0.002	
Marital status						
Married	1.80 ± 0.62	2.47 ± 1.20	1.39 ± 0.78	1.77 ± 0.79	1.26 ± 0.75	<0.0001
Single	1.97 ± 0.42	2.82 ± 1.19	1.15 ± 0.34	2.16 ± 1.18	1.33 ± 0.59	0.0003
Divorced	2.36 ± 0.54	2.88 ± 0.76	1.15 ± 0.34	3.16 ± 1.15	1.80 ± 0.61	0.0021
Widow	1.71 ± 0.61	2.43 ± 1.12	1.53 ± 1.01	1.50 ± 0.67	1.10 ± 0.42	0.0004
*p* value	0.197	0.615	0.129	<0.0001	0.268	
Level of education						
Illiterate	1.86 ± 0.64	2.74 ± 1.25	1.44 ± 0.87	1.67 ± 0.75	1.27 ± 0.84	<0.0001
High secondary school or less	1.75 ± 0.60	2.26 ± 1.09	1.37 ± 0.71	1.83 ± 0.83	1.26 ± 0.70	<0.0001
Postgraduate education (master or above)	1.80 ± 0.50	2.50 ± 1.18	1.28 ± 0.70	1.88 ± 0.73	1.16 ± 0.41	<0.0001
Doctor	2.51 ± 1.09	3.40 ± 1.44	2.08 ± 1.28	2.07 ± 0.70	2.33 ± 1.15	0.6652
*p* value	0.165	0.273	0.350	0.073	0.015	
Socioeconomic status						
Low (less than 5000 SR)	1.94 ± 0.72	2.58 ± 1.20	1.60 ± 0.99	1.91 ± 0.89	1.39 ± 0.97	<0.0001
Medium (5000–15,000 SR)	1.72 ± 0.55	2.39 ± 1.18	1.29 ± 0.64	1.71 ± 0.75	1.18 ± 0.58	<0.0001
High (more than 15,000 SR)	2.00 ± 0.64	2.88 ± 1.19	1.43 ± 0.81	1.94 ± 0.94	1.38 ± 0.70	<0.0001
*p* value	0.047	0.019	0.011	0.296	0.189	
Employment						
Unemployed	1.99 ± 0.69	2.80 ± 1.23	1.52 ± 0.94	1.86 ± 0.86	1.43 ± 1.03	<0.0001
Employed	1.84 ± 0.72	2.51 ± 1.31	1.41 ± 0.78	1.92 ± 0.93	1.21 ± 0.51	<0.0001
Retired/housewife	1.67 ± 0.49	2.26 ± 1.08	1.31 ± 0.64	1.69 ± 0.73	1.17 ± 0.52	<0.0001
*p* value	<0.0001	<0.0001	0.038	0.001	0.004	
Smoking						
Yes	1.81 ± 0.61	2.59 ± 1.21	1.27 ± 0.61	1.90 ± 0.98	1.05 ± 0.16	<0.0001
No	1.80 ± 0.64	2.52 ± 1.24	1.40 ± 0.80	1.70 ± 0.78	1.30 ± 0.82	<0.0001
Former	1.80 ± 0.55	2.27 ± 0.99	1.46 ± 0.78	1.97 ± 0.80	1.18 ± 0.52	<0.0001
*p* value	0.105	0.293	0.263	0.007	0.565	
Physical activity						
Sedentary lifestyle	1.86 ± 0.66	2.64 ± 1.23	1.44 ± 0.83	1.74 ± 0.86	1.33 ± 0.82	<0.0001
<150 min./week	1.79 ± 0.61	2.37 ± 1.15	1.36 ± 0.73	1.88 ± 0.80	1.27 ± 0.75	<0.0001
150–300 min./week	1.64 ± 0.50	2.20 ± 1.11	1.28 ± 0.68	1.70 ± 0.67	1.05 ± 0.23	<0.0001
>300 min./week	1.47 ± 0.35	1.96 ± 0.78	1.25 ± 0.71	1.48 ± 0.33	1.06 ± 0.20	<0.0001
*p* value	0.166	0.204	0.470	0.506	0.191	
Management						
Lifestyle modification	1.44 ± 0.32	1.88 ± 1.14	1.15 ± 0.34	1.44 ± 0.59	1.07 ± 0.15	0.2851
Oral hypoglycemic drugs	1.70 ± 0.55	2.29 ± 1.12	1.38 ± 0.76	1.68 ± 0.76	1.21 ± 0.51	<0.0001
Insulin use	1.88 ± 0.53	2.69 ± 1.09	1.51 ± 0.89	1.73 ± 0.81	1.23 ± 0.72	<0.0001
Insulin and oral hypoglycemic drugs	1.86 ± 0.69	2.53 ± 1.27	1.35 ± 0.73	1.89 ± 0.85	1.32 ± 0.88	<0.0001
*p* value	0.132	0.006	0.117	0.030	0.297	
Complications						
Yes	1.92 ± 0.63	2.85 ± 1.24	1.39 ± 0.85	1.80 ± 0.82	1.29 ± 0.80	<0.0001
No	1.67 ± 0.59	2.05 ± 1.02	1.40 ± 0.70	1.75 ± 0.79	1.25 ± 0.69	<0.0001
*p* value	<0.0001	<0.0001	0.827	0.553	0.534	
Ischemic heart disease						
Yes	1.97 ± 0.64	2.72 ± 1.32	1.59 ± 1.14	1.93 ± 0.54	1.28 ± 0.54	<0.0001
No	1.76 ± 0.64	2.30 ± 1.15	1.40 ± 0.76	1.79 ± 0.83	1.30 ± 0.79	<0.0001
*p* value	0.098	0.056	0.384	0.354	0.890	
Cerebral vascular accident						
Yes	1.89 ± 0.59	2.48 ± 1.14	1.48 ± 1.04	1.94 ± 0.78	1.38 ± 0.88	0.0257
No	1.76 ± 0.64	2.30 ± 1.15	1.39 ± 0.76	1.79 ± 0.83	1.30 ± 0.79	<0.0001
*p* value	0.473	0.577	0.681	0.522	0.689	
Peripheral vascular disease						
Yes	2.36 ± 0.91	3.05 ± 1.78	2.16 ± 1.71	2.48 ± 1.28	1.29 ± 0.49	0.1408
No	1.76 ± 0.63	2.29 ± 1.13	1.39 ± 0.76	1.78 ± 0.81	1.30 ± 0.79	<0.0001
*p* value	0.104	0.266	0.249	0.018	0.708	
Retinopathy						
Yes	1.91 ± 0.62	2.86 ± 1.20	1.41 ± 0.88	1.75 ± 0.79	1.27 ± 0.80	<0.0001
No	1.70 ± 0.60	2.13 ± 1.06	1.36 ± 0.68	1.81 ± 0.82	1.24 ± 0.65	<0.0001
*p* value	<0.0001	<0.0001	0.490	0.396	0.708	
Nephropathy						
Yes	1.90 ± 0.54	2.49 ± 1.08	1.52 ± 0.93	2.07 ± 0.70	1.13 ± 0.42	<0.0001
No	1.76 ± 0.64	2.30 ± 1.15	1.40 ± 0.76	1.79 ± 0.83	1.29 ± 0.79	<0.0001
*p* value	0.135	0.255	0.332	0.015	0.021	
Neuropathy						
Yes	1.91 ± 0.52	2.94 ± 1.12	1.33 ± 0.78	1.78 ± 0.78	1.17 ± 0.55	<0.0001
No	1.76 ± 0.65	2.30 ± 1.15	1.40 ± 0.77	1.78 ± 0.84	1.29 ± 0.80	<0.0001
*p* value	0.015	<0.0001	0.401	0.981	0.068	
Dyslipidemia						
Yes	1.87 ± 0.65	2.62 ± 1.20	1.38 ± 0.82	1.87 ± 0.85	1.29 ± 0.82	<0.0001
No	1.74 ± 0.61	2.27 ± 1.18	1.41 ± 0.77	1.72 ± 0.79	1.28 ± 0.68	<0.0001
*p* value	0.025	0.002	0.658	0.060	0.882	
Hypertension						
Yes	1.84 ± 0.64	2.57 ± 1.24	1.43 ± 0.82	1.78 ± 0.80	1.24 ± 0.73	<0.0001
No	1.74 ± 0.60	2.31 ± 1.16	1.38 ± 0.76	1.77 ± 0.82	1.25 ± 0.69	<0.0001
*p* value	0.125	0.028	0.524	0.956	0.854	
Family history						
Yes	1.83 ± 0.62	2.58 ± 1.20	1.38 ± 0.78	1.78 ± 0.80	1.26 ± 0.75	<0.0001
No	1.69 ± 0.62	2.04 ± 1.01	1.38 ± 0.73	1.84 ± 0.86	1.31 ± 0.72	<0.0001
*p* value	0.052	<0.0001	0.9848	0.521	0.581	
Severe hypoglycemia						
Yes	1.90 ± 0.67	2.61 ± 1.23	1.50 ± 0.90	1.81 ± 0.79	1.37 ± 0.94	<0.001
No	1.74 ± 0.58	2.39 ± 1.16	1.32 ± 0.69	1.77 ± 0.83	1.19 ± 0.56	<0.0001
*p* value	0.006	0.040	0.018	0.565	0.016	
Hospital admission						
Yes	2.04 ± 0.68	2.91 ± 1.33	1.52 ± 1.00	1.93 ± 0.79	1.47 ± 0.1.03	<0.0001
No	1.76 ± 0.59	2.39 ± 1.15	1.37 ± 0.73	1.75 ± 0.79	1.22 ± 0.66	<0.0001
*p* value	0.001	<0.0001	0.200	0.061	0.043	

**Table 9:** Partial correlation adjusting for gender, marital status, level of educations, socioeconomic status, employment, treatment modalities, and exercise.

Clinical/laboratory variables		*T*-score	*E*-score	PR-score	RR-score	IP-score
Triglyceride	Pearson coefficient (*r*)	−0.079	−0.112	−0.004	−0.025	−0.015
	*p* value	0.132	0.032	0.943	0.634	0.774
Cholesterol	Pearson coefficient (*r*)	0.023	−0.027	0.026	0.051	0.029
	*p* value	0.674	0.622	0.637	0.355	0.591
HDL	Pearson coefficient (*r*)	−0.089	−0.092	−0.066	−0.027	−0.052
	*p* value	0.115	0.104	0.241	0.638	0.360
LDL	Pearson coefficient (*r*)	0.046	0.004	0.026	0.042	0.081
	*p* value	0.404	0.942	0.639	0.444	0.138
Microalbuminuria	Pearson coefficient (*r*)	0.023	0.068	−0.057	0.025	−0.056
	*p* value	0.701	0.250	0.337	0.670	0.345
HabA1c	Pearson coefficient (*r*)	0.118	0.106	0.079	0.072	0.032
	*p* value	0.029	0.050	0.148	0.185	0.560
BMI	Pearson coefficient (*r*)	0.079	0.097	−0.024	0.070	0.018
	*p* value	0.102	0.043	0.621	0.142	0.704
Duration of diabetes in years	Pearson coefficient (*r*)	0.092	0.098	0.150	−0.037	−0.040
	*p* value	0.048	0.032	0.001	0.417	0.383
Interval between visits	Pearson coefficient (*r*)	0.159	0.115	0.061	0.165	0.078
	*p* value	0.001	0.017	0.205	0.001	0.104
Age	Pearson coefficient (*r*)	−0.029	−0.028	0.063	−0.080	−0.001
	*p* value	0.537	0.545	0.168	0.078	0.990
